# DNA polymerase III protein, HolC, helps resolve replication/transcription conflicts

**DOI:** 10.15698/mic2021.06.753

**Published:** 2021-05-06

**Authors:** Susan T. Lovett

**Affiliations:** 1 Department of Biology and Rosenstiel Basic Medical Sciences Research Center, Brandeis University, Waltham, MA.

**Keywords:** DNA polymerase, RNA polymerase, transcription termination, Rho

## Abstract

In *Escherichia coli,* DNA replication is catalyzed by an assembly of proteins, the DNA polymerase III holoenzyme. This complex includes the polymerase and proofreading subunits, the processivity clamp and clamp loader complex. The *holC* gene encodes an accessory protein (known as χ) to the core clamp loader complex and is the only protein of the holoenzyme that binds to single-strand DNA binding protein, SSB. HolC is not essential for viability although mutants show growth impairment, genetic instability and sensitivity to DNA damaging agents. In this study we isolate spontaneous suppressor mutants in a *holC*Δ strain and identify these by whole genome sequencing. Some suppressors are alleles of RNA polymerase, suggesting that transcription is problematic for *holC* mutant strains, and of *sspA*, stringent starvation protein. Using a conditional *holC* plasmid, we examine factors affecting transcription elongation and termination for synergistic or suppressive effects on *holC* mutant phenotypes. Alleles of RpoA (α), RpoB (β) and RpoC (β') RNA polymerase holoenzyme can partially suppress loss of HolC. In contrast, mutations in transcription factors DksA and NusA enhanced the inviability of *holC* mutants. HolC mutants showed enhanced sensitivity to bicyclomycin, a specific inhibitor of Rho-dependent termination. Bicyclomycin also reverses suppression of *holC* by *rpoA, rpoC* and *sspA*. An inversion of the highly expressed *rrnA* operon exacerbates the growth defects of *holC* mutants. We propose that transcription complexes block replication in *holC* mutants and Rho-dependent transcriptional termination and DksA function are particularly important to sustain viability and chromosome integrity.

Conflicts between transcription and replication are intrinsic to all cells because DNA serves as the template for both RNA and additional copies of DNA. How cells deal with this ancient problem is a fundamental question in biology.

The well-studied bacterium *E. coli* employs a strategy to replicate as quickly as possible when nutrients are plentiful. Its replicative DNA polymerase, DNA pol III, is an impressive machine, synthesizing about 1,000 nucleotides per second, bidirectionally from a single origin of replication. To divide faster than the time it takes to replicate the chromosome (roughly 50 minutes), *E. coli* initiates additional rounds of replication before completion of the previous one, such that in rich nutrient growth media, *E. coli* may have as many as four sets of bidirectional forks at any one time.

In addition, fast growth requires lots of biosynthetic capacity, especially ribosomes, with over 70,000 ribosomes per *E. coli* cell under conditions when cell division occurs every 20 minutes. Stable RNA, the bulk of which is rRNA, accounts for 98% of the RNA within an *E. coli* cell. During fast growth, each rRNA operon (of which *E. coli* has seven) initiates transcription over once per second, with rRNA transcribed at a rate of 85 nucleotides per second, considerably slower than DNA pol III. Therefore, RNA polymerase (RNAP) and DNA pol III collisions are inevitable, especially at rRNA loci that are occupied densely by elongating RNAP.

*E. coli* DNA pol III achieves high processivity by its interaction with a clamp—the clamp and the proteins that load it, a pentameric ATPase complex, are conserved in all three domains of life and essential for viability. *E. coli* has two additional accessory proteins to the clamp-loader, HolC (χ) and HolD (ψ), that co-purify as part of the DNA pol III holoenzyme but their role in replication has not been entirely clear—they are neither essential for clamp-loading *in vitro* nor essential for viability *in vivo*. We identified HolC as a gene that promoted genomic stability and tolerance to replication inhibitors, implicating a role in DNA replication fork repair.

Although HolC is “nonessential”, *holC* mutants grow slowly, especially under high nutrient conditions, and rapidly accumulate spontaneous suppressor mutations that improve viability. In this study, we sequenced the genomes of spontaneous *holC* suppressors and found a number of mutations in subunits of RNAP and a RNAP-associated protein, SspA. We then examined a number of biochemically characterized mutants of RNAP and transcription elongation factors to discern their effect on *holC* inviability.

We found that transcription initiation/elongation factor DksA sustained viability in *holC* mutants, with its loss causing synthetic lethality when combined with *holC*. On the other hand, “weak” RNAP appears to alleviate growth defects in *holC* mutants. For example, an allele of α (RpoA) that promotes RNAP instability and degradation *in vivo* was one of the original isolated *holC* suppressors. An allele of the β protein (RpoB) that causes slower elongation speed and elevated propensity for pausing and termination improves the viability of *holC* mutants. In contrast, a different allele of *rpoB* with decreased propensity for termination exacerbated the growth defects of *holC* strains. A mutation in *nusA* that reduces the efficiency of Rho-dependent transcriptional termination and the Rho-inhibitor bicyclomycin both compounded the inviability of *holC*. Bicyclomycin also negated suppression of *holC* by *rpoA* or *rpoC* alleles or loss of *sspA* (see below). Rho-dependent termination and the removal of transcription elongation complexes (TECs), then, becomes vital in HolC mutants.

Rho-dependent termination, one of two modes of transcriptional termination in *E. coli*, plays a number of important cellular roles including gene regulation, aborting transcription of untranslated genes and and limiting expression of foreign genes. In the last decade, Rho-dependent termination in *E. coli* has been linked to the maintenance of genomic stability and response to replication fork stress. Washburn, Gottesman and collaborators showed in 2011 that Rho inhibition caused replication-associated chromosome breakage and reliance on double-strand break (DSB) repair and restart proteins. Dutta, Nudler and collaborators in 2011 also observed DSBs associated with Rho inhibition and with collision of DNA pol III with back-tracked RNAP. Jain, Gupta and Sen reported in 2019 that Rho-dependent termination dislodges and recycles TECs stalled at DNA lesions. Our work indicates that in the absence of HolC, there are either more collisions of DNA pol III with stalled transcription complexes or that these collisions are more deleterious, or a combination of both.

Recently, microscopic visualization of replication components by Soubry, Reyes-Lamothe *et al.* suggests that HolC promotes excursions of components of DNA pol III (fluorescent-protein labeled DnaQ), away from the site of the replication fork (as marked by DnaB, the fork helicase) after UV-irradiation. This, and a large body of previous work, suggests that replication gaps may be left behind a progressing fork, to be filled later (**Fig. 1**). Because HolC binds to SSB, single-strand DNA binding protein, it is the likely candidate to attract DNA Pol III back to a persistent replication gap. In the absence of HolC, gaps may be slow to be filled and subject to “rear-ending” by a second fork (**Fig. 1**), causing chromosome breakage. This also explains why *holC* mutants become increasingly inviable as nutrients become more plentiful, because elevated initiation rates increase the probability of a second oncoming fork converging onto the gap.

**Figure 1:**
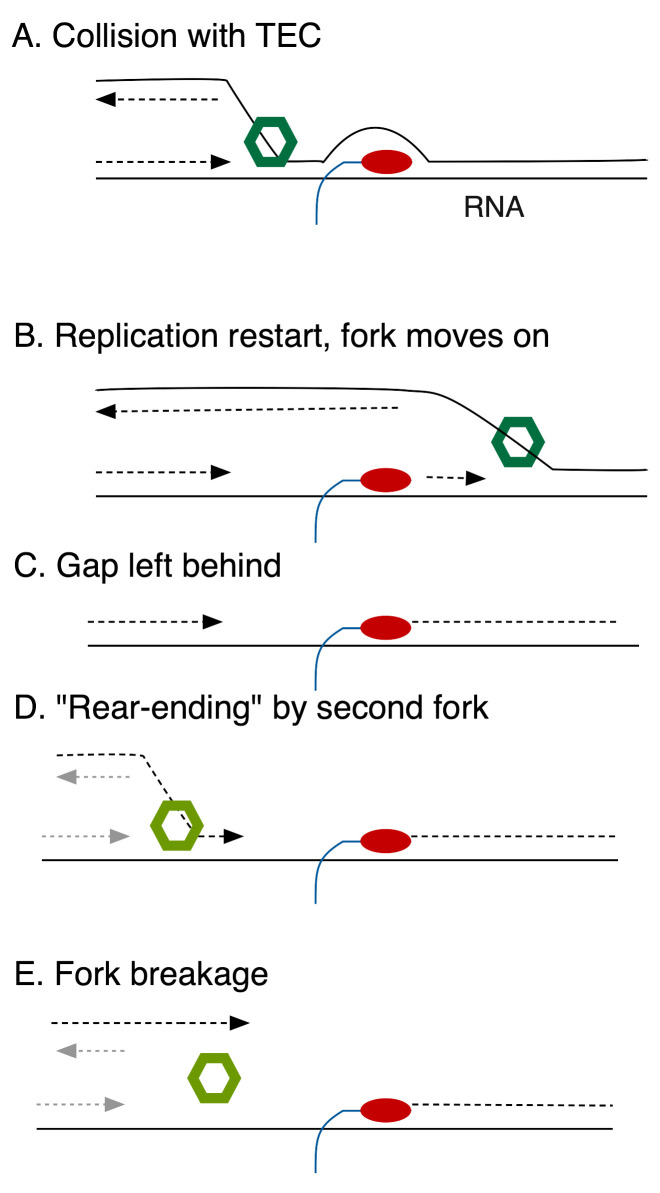
FIGURE 1: Events leading to fork breakage after collision of DNA pol III with transcription elongation complexes (TEC). Diagrammed are events ensuing from codirectional collisions, but can occur similarly with head-on collisions. RNAP is illustrated in red, the fork helicase in green. **(A)** The fork encounters a TEC, that blocks DNA polymerization. **(B)** The fork moves on, replication is restarted. **(C)** A single-strand gap is left behind. **(D)** Convergence of a second fork (helicase marked with lt green) onto the gap. **(E)** The resulting one-ended double-strand break.

Loss of function of SspA, stringent starvation protein A, ameliorated growth defects of *holC* quite dramatically, increasing the plating efficiency of *holC* mutants on rich media over four orders of magnitudes, to levels comparable to wild type strains. In 2017 Michel and Sinha reported *sspA* mutants as suppressors of the poor growth of HolD, HolC's partner protein in the clamp-loader accessory complex, likely by the same mechanism. SspA was initially identified as an RNAP-associated protein that functions as a transcriptional activator in *E. coli* and many Gram-negative bacteria, often as a heterodimer with DNA-binding proteins. The mechanism of this suppressive effect of *sspA* is unknown, but its ability to suppress *holC* is reversed by bicyclomycin suggests that it may affect Rho-dependent termination, either directly or indirectly.

Collisions of DNA pol III with TECs occur in two orientations, codirectional or head-on (**Fig. 2**). Head-on collisions are the more deleterious, possibly because TECs on the lagging strand block progression of the fork helicase, DnaB (**Fig. 2A**), whereas in codirectional collisions, the fork can proceed unimpeded (**Fig. 2B**). In *E. coli*, all seven of its highly transcribed *rrn* operons are oriented codirectionally with fork progression. Boubakri, Michel and collaborators demonstrated in 2010 that inversion of the *rrnA* operon does not kill *E. coli* but leads to dependence on a trio of DNA helicases, DinG, Rep and UvrD for viability. This same inversion becomes toxic in *holC* mutants, even with functional helicases, consistent with a role for HolC in avoidance or tolerance of head-on collisions between DNA pol III and RNAP.

**Figure 2:**
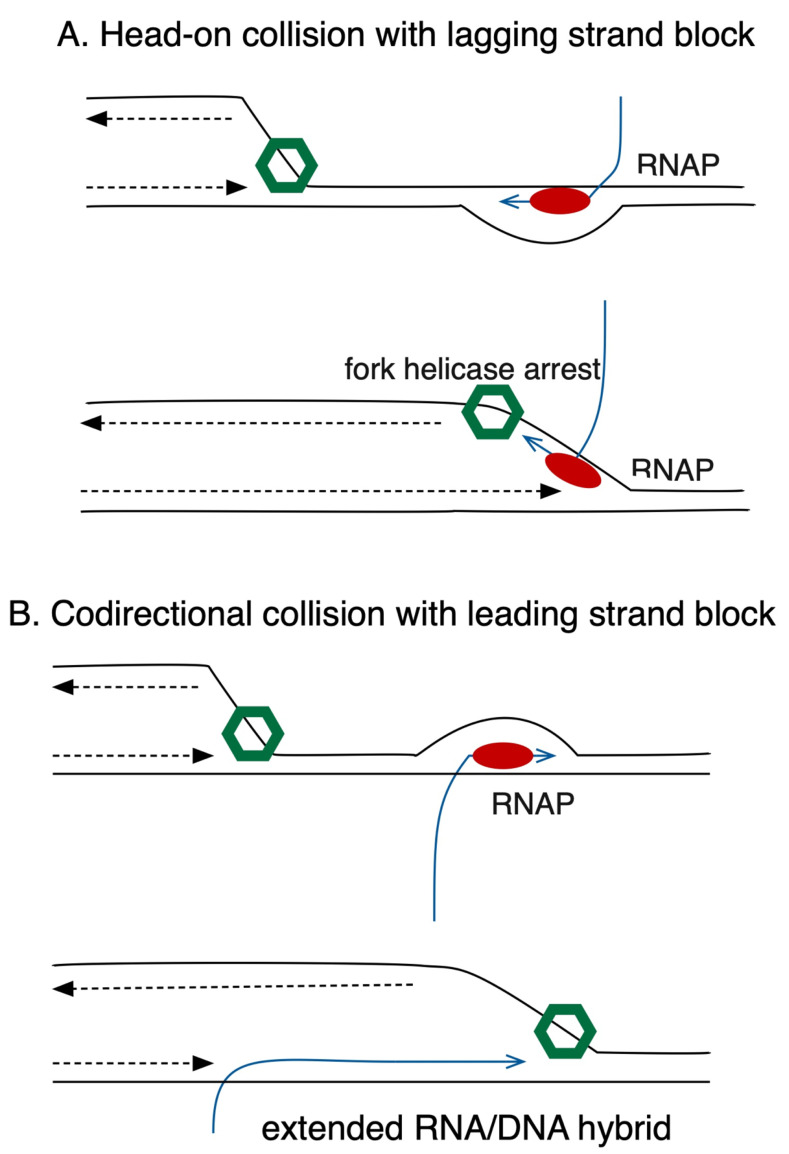
FIGURE 2. (A) Head-on collisions between the replication fork and transcription complexes. Because the DnaB fork helicase (green) translocates on the lagging strand template, head-on collisions with RNAP (red) blocks fork progression. **(B)** Codirectional collisions. DnaB fork helicase can proceed unimpeded, but fork unwinding can stabilize extensive R-loops (RNA/DNA hybrids) behind RNAP. Copyright © American Society for Microbiology, mBio12, 2021, e00184-21 doi: 10.1128/mBio.00184-21

Whether HolC affects avoidance or tolerance of co-directional collisions remains to be determined. Likewise the role of RNA/DNA hybrids (“R-loops”) and their processing needs to be explored, especially in the context of co-directional collisions, in which they might be stabilized (**Fig. 2**). The mechanism by which DksA supports survival of replication fork stress also remains unknown and will require further study.

